# Recognizing the role of the vagus nerve in depression from microbiota-gut brain axis

**DOI:** 10.3389/fneur.2022.1015175

**Published:** 2022-11-10

**Authors:** Chaoren Tan, Qiqi Yan, Yue Ma, Jiliang Fang, Yongsheng Yang

**Affiliations:** ^1^Institute of Acupuncture and Moxibustion, China Academy of Chinese Medical Science, Beijing, China; ^2^Institute of Basic Theory for Chinese Medicine, China Academy of Chinese Medical Science, Beijing, China; ^3^Guang'anmen Hospital, China Academy of Chinese Medical Sciences, Beijing, China

**Keywords:** vagus nerve, depressive disorder, gut microbiota, microbiota-gut-brain axis, gut permeability

## Abstract

Depression is a worldwide disease causing severe disability, morbidity, and mortality. Despite abundant studies, the precise mechanisms underlying the pathophysiology of depression remain elusive. Recently, cumulate research suggests that a disturbance of microbiota-gut-brain axis may play a vital role in the etiology of depression while correcting this disturbance could alleviate depression symptoms. The vagus nerve, linking brain and gut through its afferent and efferent branches, is a critical route in the bidirectional communication of this axis. Directly or indirectly, the vagus afferent fibers can sense and relay gut microbiota signals to the brain and induce brain disorders including depression. Also, brain changes in response to stress may result in gut hyperpermeability and inflammation mediating by the vagal efferents, which may be detrimental to depression. Notably, vagus nerve stimulation owns an anti-inflammatory effect and was proved for depression treatment. Nevertheless, depression was accompanied by a low vagal tone, which may derive from response to stress and contribute to pathogenesis of depression. In this review, we aim to explore the role of the vagus nerve in depression from the perspective of the microbiota-gut-brain axis, highlighting the relationship among the vagal tone, the gut hyperpermeability, inflammation, and depression.

## Introduction

Depression is a leading mental disorder causing severe disability, morbidity, and mortality worldwide. Approximately 280 million people in the world have depression (1), with almost one in five people suffering from one episode at some point in their lifetime (2). Of the leading causes of Years Lived with Disability, depression was ranked second, the highest within mental disorders, and 13th among the top 25 leading causes of Disability Adjusted Life Years in 2019, which had increased from 1990 to 2019 (1, 3). The prevalence of depression was one of the major underlying reasons of self-harm and most depressive patients have suicidal thoughts (4). According to data from WHO, there are 785,000 suicides annually around the world, with an incidence of 10.6 per 100,000 population in 2016 (5), and up to 60% of them have major depression (6).

Conventionally, depression is viewed as a mental disease with physiological dysbiosis such as unbalanced neurotransmitters and neuronal circuitry, impaired neurogenesis, declined neuroplasticity, and neuroinflammation (7–9). Recently, growing studies identified the detrimental role of disturbed microbiota-gut-brain axis in the etiology of depression and that strategies targeting to restore the balance of this axis, namely probiotics, exert a curative effect on depression (10–12). The gut microbiota (GM), the gut, and the brain interact with one another reciprocally through various routes including the vagus nerve (VN) (13, 14). The VN, a mixed nerve composed of 80% afferent and 20% efferent fibers, is able to sense and relay GM signals to the brain *via* its afferents and also, deliver brain signals to peripheral organs including the gut *via* the efferents, modulating gut dysbiosis (14). A disturbed GM led to depression-like behaviors, which may be mediated by the VN (15). Inflammation is a crucial contributor to depression (16). Vagus nerve stimulation (VNS), approved for the treatment of treatment-resistant depression and refractory epilepsy, can induce anti-inflammatory cytokines and dampen inflammation (17, 18). Besides, stimulating the VN also protects against gut hyperpermeability, which may facilitate GM translocation and result in gut and system inflammation (19). Meanwhile, a depressed vagal tone was found in depressive patients (20). It is hypothesized that through the microbiota-gut-brain axis (MGBA), a low vagal tone-induced gut barrier deficit and system inflammation may have a close relation to depression. In this review, we aim to explore the role of the VN in depression from the perspective of MGBA, putting an emphasis on the relationship among vagal tone, gut hyperpermeability, inflammation, and depression.

## Anti-depressive effect of VNS

Massive clinic research demonstrated the efficacy and safety of VNS in treating depression (C et al., 2008) (21, 22). In one multi-center study, 30 treatment-resistant depression patients with the median length of the current major depressive episode being 4.7 years, were recruited and results showed that using a ≥50% reduction in the baseline 28-item Hamilton Depression Rating Scale total score to define response, a 40% response rate along with sustained symptomatic responses were achieved after 10 weeks of VNS (23). Though another randomized, controlled acute phase trial showed tolerated results that after 10 weeks adjunctive VNS or sham treatment, 24-item Hamilton Rating Scale for Depression response rates were 15.2% for VNS group (*n* = 112) and 10.0% for sham group (*n* = 110), for which the relative shorter treatment duration may account as response rates showed a positive correlation with treatment duration (24–26). And similar accumulated antidepressant effects of VNS as mentioned above were found in a later European open-label study, in which response rates increased from 37% at 3 months to 53% at 12 months and remission rates almost doubled from 17% at 3 months to 33% at 12 months (27). Notably, in a recent 5-year, prospective, open-label, non-randomized, observational registry study, patients with treatment-resistant depression receiving adjunctive VNS with treatment as usual showed significantly better clinical outcomes compared to patients receiving treatment as usual, including a significantly higher 5-year cumulative response rate (67.6% compared with 40.9%) and a significantly higher remission rate (cumulative first-time remitters, 43.3% compared with 25.7%) (28). Intriguingly, to investigate dose-effect of VNS, another prospective, randomized, controlled trial conducted in 2013, in which participants were split into three groups: low (0.25 mA current, 130 μs pulse width), medium (0.5–1.0 mA, 250 μs), or high (1.25–1.5 mA, 250 μs), showed that though improvements of the medium and high-dose groups were better sustained at the end of the longer-term phase, the low group also had significant improvement in depressive symptoms at the end of the acute phase, suggesting that issues may exist when using low-dose VNS as a sham treatment because even low-dose VNS provided a substantial antidepressant effect (29). Besides, VNS treatment also showed promising therapeutic effects on conditions with comorbid depression including epilepsy (30), obesity (31), and migraine (32) and was capable of improving cognitive performance of treatment-resistant depression patients (33). Despite these benefits on depression and comorbid disorders, the high cost and invasiveness of VNS hampered its application and developments, both from a research and clinical perspective, and the field of a new, non-invasive VNS technique, transcutaneous auricular vagus nerve stimulation (taVNS), has drawn growing attention (34). Though in its infancy, several clinical studies had been conducted concerning taVNS in treating depression and all showed that taVNS alleviated depression symptoms with modest side effects reported (35–39). However, though evidence from various studies indicates that VNS may partly reverse the deficiency of monoaminergic neurotransmitters in the brain, peripheral and central inflammation, dysfunction of the HPA axis, abnormal neuroplasticity and neurogenesis, and disorder of functional connectivity which are implicated in depression, the antidepressant mechanism of VNS remains elusive and further efforts are warranted (2, 40–43).

## Linking of the microbiota-gut-brain axis: The vagus nerve

### Anatomy of the vagus nerve in the microbiota-gut-brain axis

Composed of 80% afferent and 20% efferent fibers, the VN transmits information and provides feedback by innervating different visceral organs including gastrointestinal tract, respiratory, and cardiovascular systems (44, 45). The gut is innervated by the hepatic and celiac branches of the VN with cell bodies of afferent neurons residing at nodose ganglia and terminating at the nucleus tractus solitarius (NTS), while the dorsal motor nucleus of the vagus nerve (DMNV), the origin of the efferent, is in close contact with the NTS (46, 47). In the gut, vagal afferents (VA) form three connections which end in muscle wall, mucosa, and of note, the recently defied neuropod cells which are a subset of enteroendocrine cells synapsing with vagal neurons (48). Attributing to their locations and a variety of receptors including mechanoreceptors and chemoreceptors expressed on them, VA are capable of detecting and responding to various sorts of signals including stretch, tension, or intestinal molecules such as bacterial by-products, gut hormones, or neurotransmitters (19, 49).

In the brain, the NTS together with the DMNV, form an autonomic brainstem loop regulating gastrointestinal motility, acid secretion, food intake, and satiety (50). Through projections from the NTS to several regions of the central nervous system (CNS), including the parabrachial nucleus, paraventricular nucleus of the hypothalamus, locus coeruleus, the amygdala, and the thalamus, the NTS form an autonomic brainstem loop which is modulated by the autonomic forebrain loop which comprises nuclei in the pons, the hypothalamus, the hippocampus, the amygdala, the anterior cingulate, the insular, and the prefrontal cortices, synthetically coordinating visceral information that includes neuroendocrine responses, emotions, cognition, and behaviors (50). Thus, the brain is in close communication with the gut through these two loops and the VN and stress, feelings, and thoughts may play a beneficial or detrimental role in gut homeostasis and vice versa.

### From gut microbiota to brain: Role of vagus

Though the VN is unable to reach luminal contents, it can indirectly sense gut information through communication with enteroendocrine cells (EECs) (51). By releasing mediators including serotonin, cholecystokinin, glucagon-like peptide-1, and peptide YY, acting on corresponding receptors expressed on vagal afferent neurons, the EECs can modulate food intake and autonomic reflexes controlling gut motility, secretion, inflammatory responses, and mucosal defense (52). And the latest identified neuropods have demonstrated that apart from paracrine and endocrine, the EECs have the capability of forming synapses with neurons of the vagal nodose to transduce a sense from gut to brain with glutamate, to our knowledge, as the neurotransmitter (53). Most pivotally, as the first cell line facing the gastrointestinal contents, the EECs can detect signals from GM which may in turn influence the releasing of chemical stimuli including hormones, neurotransmitters, and metabolites produced by EECs (54, 55). Indeed, both *in vitro* and *in vivo* studies demonstrated that the EECs expressing toll-like receptors (TLRs) can sense microbe-associated molecular patterns (MAMPs) including lipopolysaccharide (LPS) and flagellin, which are constitutively released by bacteria (56, 57). In addition to secreted bacterial ligands by microbes, the EECs can also detect bacterial metabolites (58, 59). For example, short-chain fatty acids (SCFAs) can bind to G protein-coupled receptors (GPCRs) carried on EECs, including GPR40, GPR41, GPR43, GPR119, and GPR120, and in turn, stimulate the VN, regulating host metabolism and feeding behavior (60, 61). And in response to indole, the colonic enteroendocrine L-cells can secrete glucagon-like peptide-1 to stimulate colonic VA activity (62). Furthermore, certain microbes can directly infect enteroendocrine cells and upregulate glutamate transporters, indicating that these affected cells may be neuropods, directly synapsing with VA (63, 64). Taken together, these findings suggest that the EECs are a crucial interface for detecting GM signals and relaying these massages from gut luminal to the CNS *via* VA.

Apart from interchanging with EECs, another pathway transmitting intestinal canal information to the CNS relies on innervation of the enteric nervous system (ENS) by the VN (65). The ENS, known as the “second brain of the body,” is crucial for maintaining stable gut health, which requires both efforts from enteric neurons and the connections to the CNS, namely the VN and sympathetic nerves (66). Located in either the submucosal or myenteric plexus, enteric neurons, with mechanoreceptors and chemoreceptors, are responsive to molecular and mechanical aberrations of the gastrointestinal tract and, in turn, activate vagal afferent neurons (67). For example, the microbiota affect both developments and function of the ENS by activating pattern recognition receptors, including TLRs, especially, TLR2 and TLR4 (68). Indeed, intrinsic primary afferent neurons were activated by ingestion of *Lactobacillus rhamnosus* (JB-1) and in turn stimulated part of the VA (69). Other microbiota metabolites have also been shown to influence the ENS activity and regulated gut motility in rodents (70). In addition, by delivering neurotransmitters and neuropeptides, intrinsic neurons of the ENS can modulate immune cells and cytokines produced by immune cells which have a reciprocal effect on neurons (67).

Directly, the VA also express TLR4 and free fatty acid receptor 3, sufficiently detecting MAMPs and SCFAs by themselves (71, 72). TLR4 mRNA and protein was expressed in the rat nodose ganglion, suggesting that administration of LPS could activate VA at the level of nodose ganglion (73). Owing to the gut barrier, it seems unlikely for these neurons to be directly contacting these MAMPs in physiological condition. Nevertheless, once the integrity of this barrier is lesioned under circumstances such as confronting stress, burn injury, or gastrointestinal disorders, including irritable bowel syndrome (IBS) and functional constipation, chances for the translocation of GM and their metabolites crossing the gut epithelial wall soar, with upregulation of 5-hydroxytryptophan, over expression of the corticotropin releasing factor (CRF), excessive TLR activation, and decreasing level of SCFAs being the potential contributors for this deleterious process (74–80). The gut immune system is activated by antigens from intestinal lumen and consequently, immune active substances including cytokines are released by immune cells such as T cells, B cells, innate lymphoid cells, macrophages and dendritic cells (81, 82). For instance, when activated in response to pathogens and other injurious stimuli, macrophages, dendritic cells, and other cells in the mucosa can produce a cytokine named tumor necrosis factor alpha (TNF-α)(83, 84). When exposed to cytokines including TNF and interleukin-1 beta (IL-1β), VA from nodose ganglion expressing cytokine receptors sense the peripheral inflammation condition and propagate action potentials to the CNS which then activates anti-inflammation responses including the cholinergic anti-inflammatory pathway (85, 86).

**Figure 1:**
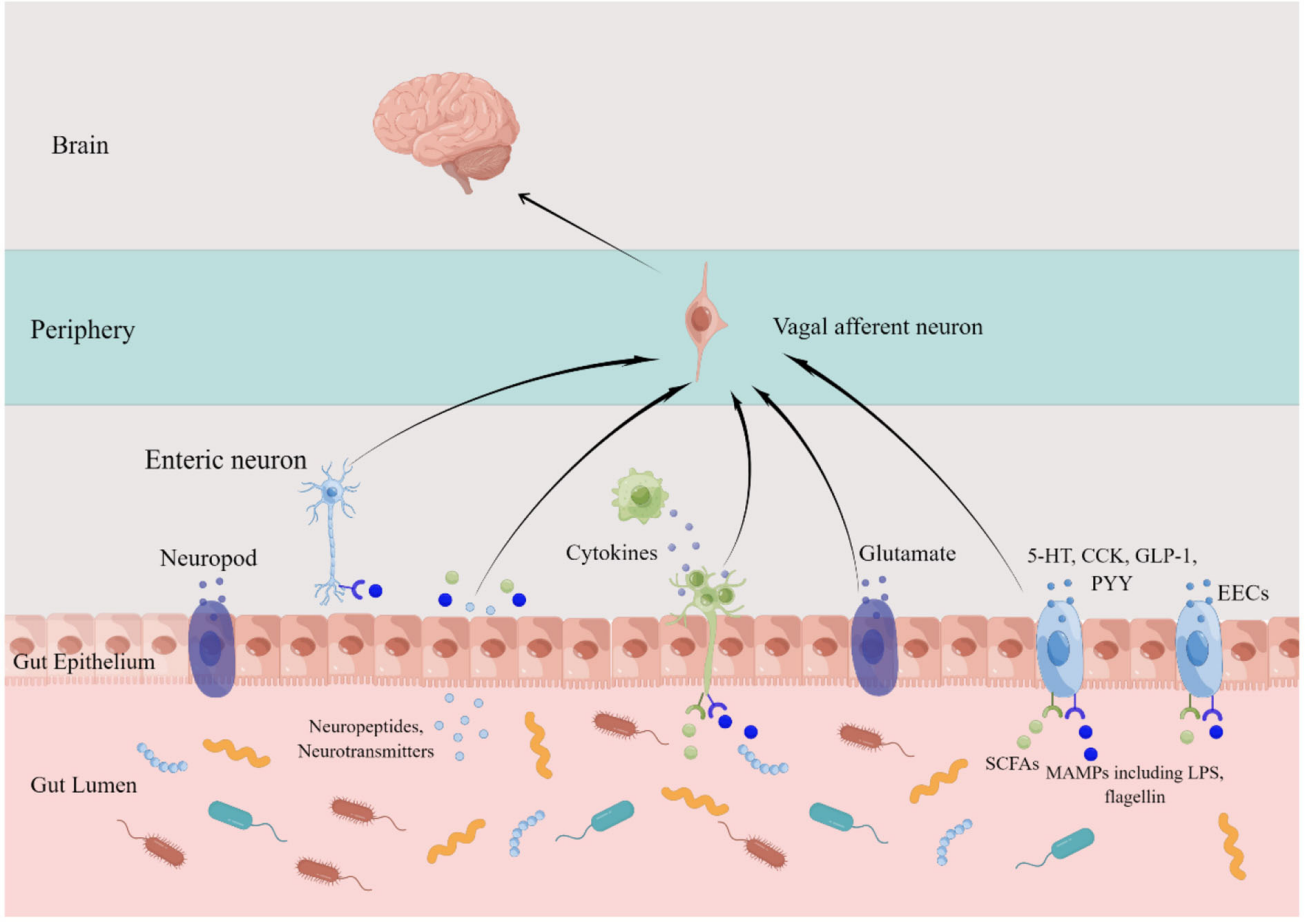
Vagal afferents transmit signals from gut microbiota to the central nervous system. 5-HT, serotonin; CCK, cholecystokinin; GLP-1, glucagon-like peptide-1; PYY, peptide YY; EECs, enteroendocrine cells; SCFAs, short-chain fatty acids; MAMPs, microbe-associated molecular patterns; LPS, lipopolysaccharide. By Figdraw.

Besides, certain species of intestine bacteria have the capacity to synthesize multiple neurotransmitters including γ-aminobutyric acid (GABA), noradrenaline, dopamine, and serotonin which can travel through portal circulation to affect the afferent pathway of the VN (87, 88). For example, when the culture pH condition was adjusted to the optimal pH of glutamate decarboxylase activity, JB-1 could produce GABA with a supplement of monosodium glutamate and pyridoxal phosphate and ingestion of *Lactobacillus* strain induced GABA receptors alteration in a region-dependent manner in the brain which were not observed in vagotomized mice (89, 90) (Figure 1).

Indeed, using an *ex vivo* intestinal jejunal segment mesenteric nerve recording preparation, Perez-Burgos et al. have shown that intra-luminally adding JB-1 could increase the firing rate of VA and these neurons may partially function as interneurons for stimulation by JB-1, receiving input from intrinsic primary afferent neurons, which synapse with VA and can be also activated by JB-1 (69, 91, 92).

Above all, on the one hand, the VA indirectly perceive GM signals through mutual communication with EECs, ENS, and the gut immune system, which interact with one another, with bioactive substances like neurotransmitters, neuropeptides, cytokines, and hormones as mediators. On the other hand, the GM and their metabolites, including SCFAs and neurotransmitters, may directly activate the VA. Together, these two pathways constitute the vagal afferent limb transmitting GM messages to the CNS, which then integrates these viscera messages and sends feedback to regulate gut homeostasis.

## The microbiota-gut-brain axis and depression

### Altered intestine microbiota composition in depression

In both clinical and animal models, the GM of depressed patients and animals with depressive behaviors were found to be significantly different from that of healthy controls. In depressive patients, both microbiota richness and diversity were declined in some studies (93, 94). Though there was no difference of gut microbiota composition in α-diversity in the majority of the major depressive disorder (MDD) studies between MDD patients and their healthy counterparts, a difference did exist in β-diversity (95, 96). In general, at the phylum level, the richness of Actinobacteria was higher in MDD while abundance of Bacteroidetes was lower; at the family level, the relative abundance of *Bifidobacteriaceae*, and *Lachnospiraceae* and *Prevotellaceae* was higher and lower, respectively; At the genus level, the abundance of *Sutterella* and *Faecalibacterium* was decreased, while that of *Eggerthella, Olsenella, Collinsella, Lactobacillus, Oscillibacter* increased (95). Even though distinctions of GM were observed between patients and controls in these studies, it is still debatable to determine the specific microbiota taxa which have the capacity to draw a line between the two groups (97, 98), and this uncertainty may be due to several factors such as psychiatric medication status, type of comparison group, medical comorbidities, and detection methods of fecal microbiota (99). Further research aiming to seek out the marker bacteria species that precisely discriminate the depressed patients and healthy individuals, moreover, even different types or periods of depression, are warranted to take these factors into account.

Animals with induced depressive behaviors also presented a loss of microbiota richness and diversity. The disturbance of GM composition was found in different animal models including the maternal separation model, chronic unpredictable stress model, chronic social defeat model, chronic restraint model, and the bilateral olfactory bulbectomy model (100–104). However, alterations of GM in animals with depression-like behaviors were different from that of depressed patients, with some changes even to be in the opposite direction, for example, the abundances of Bacteroidetes were lower in patients while increased in animals (101, 102).

Though we know that depressed patients are usually accompanied by disturbed GM, how this altered GM evolves and its role in depression warrant further investigations.

### Fecal microbiota transplantation and depression

Depression, beyond our conventional appreciation of being a non-infectious neuropsychological disease, is transmissible through fecal microbiota transplantation (FMT) from depressed donors, either patients or animals, to recipient. Julie found that Flinders Resistant Line rats receiving FMT from MDD patients displayed significantly more depressive-like behaviors than those receiving FMT from healthy individuals (105). Moreover, intestine microbiota taxa were also transferred to recipients with the group receiving FMT from MDD patients carrying on certain taxa which was similar to their donors. The same results were found in another model in which recipient rats were depleted microbiota *via* an antibiotic cocktail treatment and presented depression-like symptoms including anhedonia and anxiety-like behaviors, as well as alterations in tryptophan metabolism, all of which resembled those of their counterpart providers (93). Besides, FMT from depressed animals to healthy recipients also induced depression phenotype, consistent with that of patients (106, 107). Fascinatingly, a recent study found that ingestion of FMT from rheumatoid arthritis patients resulted in the same depression-like phenotypes in antibiotic cocktail-treated mice (108). This transmissible characteristic of depression through FMT puts forward the hypothesis that a disturbed GM may play a causal rather than consequent role in its relationship with depression. However, more valid evidence is warranted to confirm this assumption.

Interestingly, in addition to these “depression-related microbiota,” some research indicated that the FMT of healthy individuals may possess a therapeutic effect. Kishimoto found that patients with either IBS, functional diarrhea, or functional constipation who received FMT of healthy relatives within the second degree of relationship (≥20 years of age) showed an improvement of depression and anxiety symptoms regardless of gastrointestinal symptom change (109). Other studies showed that both the gastrointestinal symptoms and depressive behaviors were alleviated after FMT, with this effect persisting over 4 weeks (110, 111).

Overall, it is possible for depression to transmit through fecal microbiota, though this is unlikely to happen in natural conditions. FMT may be effective adjunctive therapy in treating gastrointestinal or psychiatric disorders including IBS and depression, nevertheless, further FMT studies are worth pursuing to determine its efficacy and security.

### Probiotics alleviate depression symptoms

Probiotics are generally defined as “live microorganisms that, when administered in adequate amounts, confer a health benefit on the host” (112). Beyond functioning locally in the gastrointestinal tract and treating gastrointestinal disorders including inflammatory bowel disease (IBD) (113) and IBS (114), more studies found that administration of probiotics can influence brain function and cure mental disorders (115) including Alzheimer's disease, Parkinson's disease, and depression, and the probiotics possessing beneficial effect on patients suffering from mental health issues are named psycho-biotics, first introduced by Dinan et al. (116). Indeed, clinical and animal studies have shown that probiotics supplementation could alleviate depression symptoms. In randomized, double-blind, placebo-controlled studies, probiotics significantly improved depression symptoms of depressive patients, though inconsistent results were reported concerning improvement of anxiety symptoms and serum inflammation markers (117–119, 193). Animal studies indicated that the anti-depressive effect of probiotics may attribute to mitigating gut microbial dysbiosis (120–122, 195), and this effect may not require the bacteria to be alive (112), suggesting a vital role of bacteria components in the effect. The most reported probiotics belong to lactic acid bacteria and *Bifidobacterium*, including strains JB-1 (123), *Lactobacillus gasseri* (124), Bifidobacterium longum NCC3001 (118) and *Bifidobacterium breve* CCFM1025 (112) and recently recognized *Akkermansia muciniphila* (121).

## The vagus nerve plays a vital role linking gut microbiota to depression: Insight into the subdiaphragmatic vagotomy studies

Recent studies identified the essential role of the subdiaphragmatic VN in the transmission of depression through FMT. Wang et al. found that ingestion of fecal microbiota from chronic social defeated stress susceptible mice induced an anhedonia-like phenotype, higher plasma levels of interleukin-6, and decreased expression of synaptic proteins in the prefrontal cortex (PFC) in antibiotic-treated wild type mice and *Ephx2* (coding soluble epoxide hydrolase) knockout (KO) mice but not in water-treated mice (125, 126). Further research found that *Lactobacillus intestinalis, Lactobacillus reuteri* and *Faecalibaculum rodentium* may, respectively, account for the anhedonia-like phenotype in antibiotic-treated wild type and *Ephx2* KO mice after FMT, as merely ingestion of these microbiota duplicated these results. In another study, FMT from *Chrna7* (coding α7 subtype of the nicotinic acetylcholine receptor, α7 nAChR) KO mice also resulted in depression-like phenotypes, systemic inflammation, and downregulation of synaptic proteins in the PFC in antibiotic cocktail-treated mice (127). Most importantly, subdiaphragmatic vagotomy significantly blocked the development of behavioral abnormalities, systemic inflammation, and downregulation of synaptic proteins in the PFC after ingestion of the three strain bacteria or receiving FMT from *Chrna7* KO mice (125–127). These provide evidence supporting the hypothesis that the disturbance of intestine microbiota may have a causal effect on depression which is mediated by subdiaphragmatic VN and selectively deafferenting VN may be a target for effectively treating depression. Besides, subdiaphragmatic vagotomy also blocked the depression-like phenotype induced by intraperitoneal injection of LPS in rats (128). Further, a recent study found that LPS administration in mice also caused abnormal composition of gut microbiota, along with a depression-like phenotype, increasing of spleen weight, triggering of systemic inflammation and downregulating of synaptic proteins in the medial PFC in a subdiaphragmatic VN-dependent way (129). Most interestingly, they found a significant increase in the relative abundance of *Lactobacillus reuteri* after LPS administration which was consistent with the study of Wang et al. (125), that found positive correlations between the plasma levels of IL-6 (or TNF-α) and spleen weight and correlations between spleen weight and the abundance of the components of the microbiome. These results indicate that first, in addition to the “down to top” (from the gut to brain) effect, the subdiaphragmatic VN also mediates the “top to down” (from the peripheral or brain to gut) effect; second, the *Lactobacillus reuteri* may play a vital role in the pathogenesis of depression; third, as the biggest immune organ, the immune effect of the spleen in response to LPS from intraperitoneal administration or possibly leaking from the gut, may account for system inflammation and depression.

In addition, subdiaphragmatic VN dependent gut-brain signaling also contributes to the effects of oral selective serotonin reuptake inhibitors (SSRIs). After chronic or acute ingestion of sertraline or fluoxetine, two kinds of SSRIs, Karen-Anne found an increase in vagal fiber activity and the intactness of vagal signaling was required for this anti-depressive effect of chronic ingestion SSRI as subdiaphragmatic vagotomy abolished this effect, determined by the tail suspension test (130). Additionally, certain probiotics also needed this intact vagal signaling to play its anti-depressive role (124). Though, the precise mechanism remains unknown, these highlight the potential for pharmacologically stimulating VN to treat mood disorders even without the agents entering the circulation.

## Vagal tone, gut permeability and inflammation, and depression

### Stress as a risk factor for depression

From an evolutionary aspect, stress is a necessary response to stimulus that activates the fight-or-flight mechanism in the body which is essential for the survival of any organism (131). Proper stress enhances the acquisition and/or expression of immuno-protective responses such as wound healing, vaccination, anti-infectious agent, and anti-tumor, while inappropriate chronic stress is related to some psychiatric and gastrointestinal diseases including depression and IBD (131–133). Indeed, life stress, notably early life adversities including emotional maltreatment, physical abuse, and sexual abuse, was proved to be a risk factor for the onset of adulthood depression, which may be mediated by continued stress (134, 135). However, some indicated that those suffered childhood adversities without an early onset, seemed to gain benefit from childhood adversities, showing resilience to depression in high-stress, which suggests that it may be effective to distinguish between various types of reaction patterns based on the age at first onset of depression (136). In animals models, those that had undergone maternal separation and/or chronic stress such as chronic unpredictable stress, chronic restraint stress, and chronic mild stress, all developed depressive behaviors which were improved by administration of probiotics or prebiotics (137).

### Compromised gut barrier function in depressed patients and stress induced depressed animals

Cumulative evidence demonstrates that stress can damage the gut barrier integrity in animals (137, 138). The HPA axis mediates the response to stress and activation of this axis results in the releasing of the CRF, which together with its receptors, mainly CRF1 and CRF2, plays a vital role in the gut permeability disturbance induced by stress exposure (139–141). Indeed, plasma zonulin and fatty acid-binding protein-2, the biomarkers of increased gut permeability, accompanied with plasma LPS, part of the bacteria wall of gram-negative bacteria, were found increased in subjects with a depressive disorder or an anxiety disorder, suggesting the existence of compromised gut epithelium barrier integrity and GM translocation in depressive patients (142). The gut hyperpermeability or the so-called leaky gut, may facilitate the translocation of GM and their metabolites through the intestinal epithelial barrier, further into the circulation system, promoting neural, endocrine, and immune responses (77). The serum immunoglobulin M and immunoglobulin A against LPS was also elevated in MDD, indicating an adaptive immune response to a gram-negative bacteria load and this may partly explain the increased relative abundance of gram positive cocci in rats that have undergone chronic early-life stress (143, 144). Further, this immune response was observed in Bipolar Disorder patients too, with these aberrations in the gut-brain axis most pronounced in Bipolar Type 1 and melancholia (145). Interestingly, the injection of LPS, even with minimal amounts, to human volunteers may decrease mood and induce anxiety (146).

### Chronic inflammation induced by gut barrier dysfunction

System and neural inflammation play a crucial role in pathogenesis of depression while how this chronic inflammation condition forms and persists in depressive patients is unclear. The immune response to GM translocation induced by leaky gut may be responsible for the chronic inflammation condition in depression. The activation of immune cells by LPS binding to TLR4 activates nuclear factor kappa-B (NF-κB), one intracellular signaling molecule, which in turn promotes the production of pro-inflammatory cytokines, including TNF-α and IL-1 and cyclo-oxygenase-2 (147, 148). The same process also induces oxidative and nitrosative stress (O&NS) pathways, increasing expression of inducible nitric oxide and production of reactive oxygen species (ROS) by further activating nicotinamide adenine dinucleotide phosphate oxidase (149). The overload of ROS not only activates NF-κB, but leads to DNA damage and cell death, both processes aggravating the inflammatory state (150). Worse yet, cytokines including interferon-α, IL-6, IL-1β and TNF-α, and O&NS pathways may cause loosening of the tight junction barrier, forming a pro-inflammatory circle between intestine hyperpermeability and host immune response (149). This circle, at least partly, accounts for the chronic low-grade but sustained inflammation state of depressive patients. Therefore, adopting strategies targeted to protect or improve intestine epithelial integrity may exert benefits to diseases associated with the compromised gut barrier including depression while those, including stress, infection, poor diet, and antibiotics, undermine this barrier intactness resulting in deterioration of these diseases.

### Gut inflammation and depression

Peripheral inflammation is closely connected to depression. Inflammation markers including serum IL-6, IL-1β, and C-reactive protein were elevated in depression patients (151, 152). Furthermore, administration of proinflammatory cytokines to humans or animals for treatment, has been found to induce depressive symptomatology, which was attenuated by treatment with antidepressants such as tricyclic antidepressants and SSRIs (153). Additionally, administration of LPS increased plasma concentration of cytokines and simultaneously induced depressive symptoms (146, 154). Most importantly, patients with gut inflammatory disorders have high co-occurrence of depressive behaviors. According to a recent Nature review, despite pronounced heterogeneity, the pooled prevalence of symptoms of depression in IBD, including Crohn's disease (CD) and ulcerative colitis, was reported over 20% (194). In addition, elevated depressive symptoms over time were associated with increased odds of active IBD (155). Besides, in studies of induced colitis in mice, behavior abnormality consistent with depression was observed, accompanied with an increase in circulating pro-inflammatory cytokines (156, 157). Interestingly, the anterior cingulate cortex (ACC), part of the autonomic forebrain loop, functioning as a relay hub and transmitting various input signals after evaluating requirements from other regions to guide adaptive behaviors, is particularly sensitive to cytokines (158, 159). Notably, both depression and CD are inflammation-related disorders and dysfunction of the ACC was reported in depression and CD (160, 161). Further, a recent study from Xu group found that patients with CD who were in the active phase exhibited higher amplitude of low frequency fluctuation (ALFF) in the left ACC and a positive correlation between mWavelet-ALFF values of the ACC and Hospital Anxiety and Depression Scale-depression scores in CD patients (162). These suggest that ACC may serve as an intersection in the brain, which senses gut inflammation and responses inappropriately, increasing risk of depression.

Indeed, through the leaky regions of the brain-blood barrier, the circumventricular organs or the neural routes, for instance the VN fibers, peripheral cytokines can reach the brain and stimulate the brain immune cells, mainly microglia (163, 164). Apart from this, immune cells like T cells, by upregulating various adhesion molecules and integrins, are attracted by chemokine to infiltrate the brain and produce cytokines in meninges or parenchyma (165). Then, the concentration of cytokines and activated microglia result in neuroinflammation, exerting detrimental effects on neurogenesis in the hippocampus which plays a direct role in the pathophysiology of depression (166–168). Also, by activation of indoleamine 2,3 dioxygenase and tryptophan 2,3 dioxygenase, enzymes catalyzing the conversion of tryptophan into N-formylkynurenine, pro-cytokines decreased tryptophan levels and increased production of kynurenine and other tryptophan-derived metabolites, resulting in less precursors for serotonin synthesis (169, 170). In addition, cytokines, in particular IL-1 through activation of p38 mitogen-activated kinase, inhibited glucocorticoid receptor function, sharing HPA-activating activity (171, 172).

### Anti-inflammatory property and epithelial barrier protective role of VNS in the gut

The VN owns an anti-inflammatory property and was first introduced by Tracey (84), called “cholinergic anti-inflammatory pathway” (CAIP) (173). Through the vago-sympathetic reflex, VNS activates the splenic sympathetic nerve which releases norepinephrine binding to the β2 adrenergic receptor of splenic lymphocytes. And the acetylcholine (ACh) delivered by splenic lymphocytes in turn binds to α7 nAChRs of splenic macrophages to finally inhibit the release of TNF-α, a pro-inflammation cytokine (174). Further, the Janus kinase 2-Signal transducer and activator of the transcription 3 signaling pathway is implicated in this anti-inflammatory effect (175). In the gut, similarly, VNS also possesses an anti-inflammatory role depending on enteric neurons. When activated by systemic inflammation or local peripheral inflammation, the VA relay inflammation signals to the NTS, which through projection to the DMNV activates vagal efferents, which then stimulate enteric neurons and, in turn, the enteric neurons activate macrophages by releasing ACh, binding to α7 nAChRs, and inhibiting TNF-α releasing (85). The anti-inflammatory property in the gut of the VN was proved in an animal model of postoperative ileus, independent of the spleen and T cells (18). This effect was also observed in a number of studies of rodent colitis, such as dextran sulfate sodium, oxazolone-, and 2,4,6-trinitrobenzene sulfonic acid-colitis (176–178). Furthermore, in a recent animal study, rats were induced a small intestinal inflammation by indomethacin and VNS reduced the small bowel inflammation in a spleen-independent mechanism, suggesting a direct anti-inflammation effect of the VN in the gut (179).

In addition to the anti-inflammatory property, VNS also displays a direct protective role in intestinal epithelial barrier integrity. Male mice were subjected to a surface area steam burn to induce intestinal epithelial barrier breakdown and intestine inflammation, both of which were attenuated by VNS, either before or after the burn insult, and this preventive effect of VNS in intestine dysfunction was also observed in the traumatic brain injury model (180–182). Latter research identified a role of α7 nAChR and activation of enteric glia cells in this effect (183, 184). Several studies also indicated that electroacupuncture could protect against intestinal hyperpermeability through the VN, as vagotomy or intraperitoneal administration of an α7 nAChR inhibitor reversed this effect (185, 186). In another mice model challenged with LPS, VNS decreased expression of tight junction proteins occludin and zonula occludens through suppressing translocation of NF-κB p65 and downregulating myosin light-chain kinase (187). However, there is no publishing data concerning the effect of VNS on the intestinal epithelial barrier in depressive patients or animal models. Further studies are warranted to determine the relation between this effect and depression.

### Linking the depression and gut inflammation: The vagal tone

**Figure 2:**
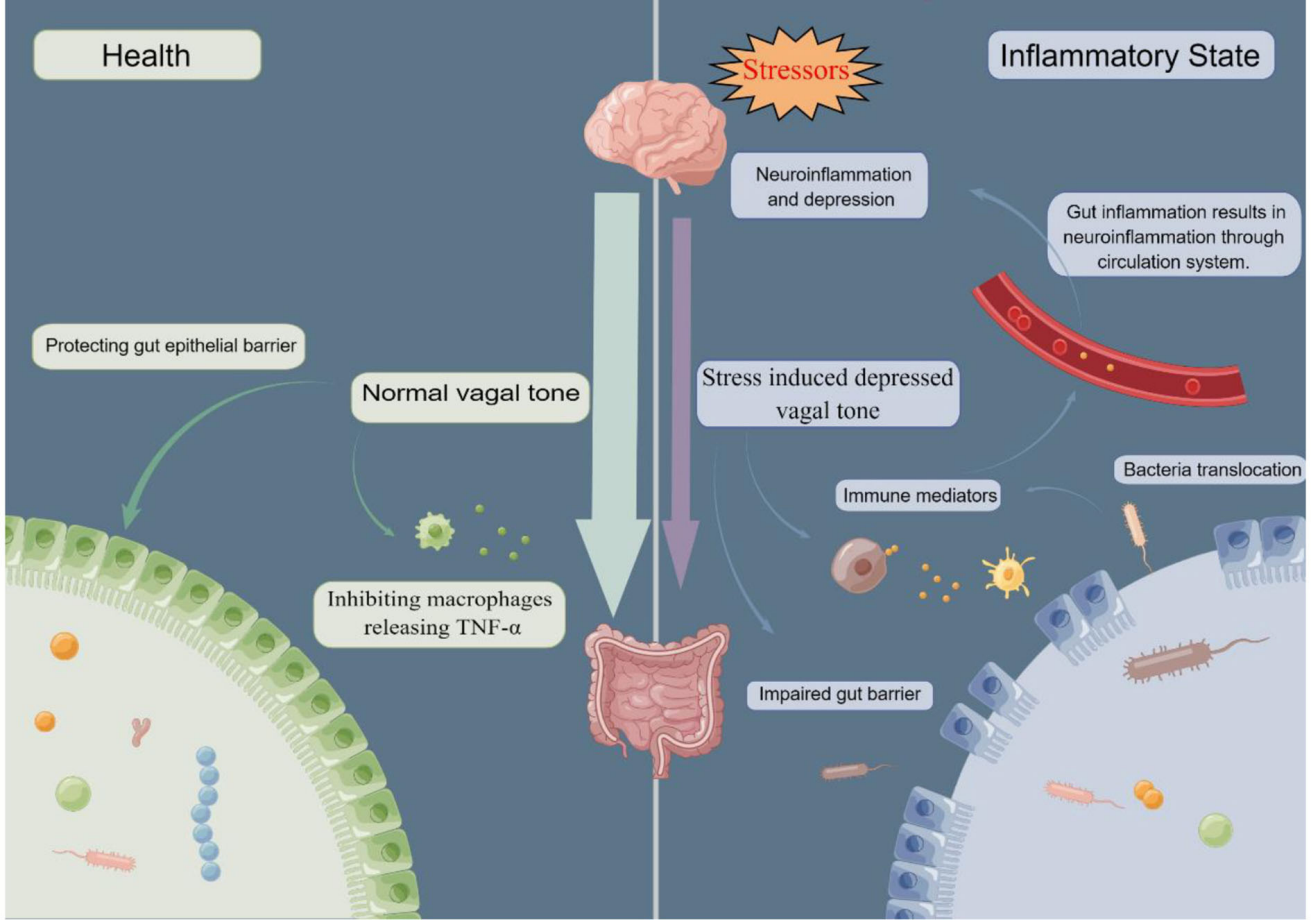
In healthy conditions, normal vagal tone can protect gut barrier and inhibit macrophages releasing pro-cytokine, TNF-α. While in response to stressors, vagal tone is depressed, promoting gut barrier impairment and releasing of TNF-α. Compromised gut barrier facilitate bacterial translocation, which activates immune system and induces immune mediators. Through the circulation system, gut and system inflammation ultimately results in neuroinflammation. TNF-α, tumor necrosis factor alpha. By Figdraw.

Importantly, stress inhibits vagal tone and decreased heart rate variability, a marker of low vagal tone, was found in depressed patients, and also in IBD (20, 188, 189). Since increasing vagal tone by VNS displays an anti-inflammation effect in the gut mediated by macrophages, impaired vagal tone including in response to stress, may lead to a pro-inflammatory state in the gut. Indeed, Ghia and colleagues reported that impaired vagal function in mice following induced depression led to gut inflammation while restoration of parasympathetic function through administration of desmethylimipramine, a tricyclic antidepressant, protected against gut inflammation (190). In addition, Ghia et al. found that macrophages isolated from vagotomized mice showed an increase of proinflammatory cytokine release including IL-1β and IL-6 (191). Further, vagotomy reactivated inflammation in mice with chronic colitis. Additionally, in a nationwide register-based matched cohort study, Liu et al. found a positive association between vagotomy and later IBD, particularly CD (192). Together, these findings suggest that stress-induced depressed vagal tone leads to a pro-inflammatory state in the gut. Thus, stress, on the one side, directly wound the gut barrier, promoting gut bacteria translocation and gut inflammation; on the other side, through inhibition of vagal tone, prime immune system induces a pro-inflammatory state and aggravates gut and system inflammation, eventually leading to depression (142) (Figure 2).

## Contribution and limitations

In this review, we discussed the relationship between MGBA and depression and the role the VN plays in this relationship, hypothesizing that increasing vagal tone by stimulating the VN, electrically or chemically, may alleviate depressive symptoms through improving gut inflammation and permeability. This hypothesis links stress (risk factor for depression), gut hyperpermeability, and inflammation (found in depressed animals and patients) and the VN, partly explaining the origin of system and neuroinflammation and their contribution to depression. However, there are several limitations. First, to put forward a self-consistent hypothesis, we selectively put inflammation in the intersection and stressed the role of the VN in inflammation-mediator-induced depression models, while in non-inflammatory depression models, the anti-depressive effect of VNS may rely on other mechanisms rather than the anti-inflammatory and gut barrier protective role of the VN. Second, though it is clear that the VN mediates the contribution of disturbance of MGBA to depression, no unanimous conclusion that dysbiosis of GM precedes depressed brain or vice versa can be drawn. In future, more research is required to probe whether in non-inflammatory depression models, stimulating VN can still reverse the dysbiosis of GM to improve depression and to determine a causal relationship between gut and brain disturbances.

## Conclusion

Depression is more than a mental disease but with substantial physiological and anatomic alterations including decreased neurogenesis and neuroplasticity, dysfunction of neural circuits, and unbalanced neurotransmitters. Disturbed MGBA was reported to be a contributor to depression and restoration of the disturbance of this axis by administration probiotics is effective in alleviating depressive symptoms. The VN is a pivotal route for bidirectional communication of MGBA, mediating the anti-depressive or pro-depressive effect of gut microbiota on depression. Nevertheless, further research is warranted to determine specific bacteria strains owning an anti-inflammatory role and its molecular basis *via* the VN. The VN possesses an anti-inflammatory and gut barrier protective role which is inhibited in response to stress, a risk factor of depression. It is likely that the depressed vagal tone may facilitate the gut bacteria translocation and system inflammation, promoting the onset and deterioration of depression. More clinic and pre-clinic studies are needed to test and improve this hypothesis.

## Author contributions

YY, QY, JF, YM, and CT conceived the idea and revised extensively this review. QY, YM, and CT searched the literature. CT drafted the manuscript. All authors approve the final manuscript prior to submission.

## Funding

This work was supported by China Academy of Chinese Medical Science Innovation Fund (No. CI2021A03301) and National Natural Science Foundation of China (No. 81273833).

## Conflict of interest

The authors declare that the research was conducted in the absence of any commercial or financial relationships that could be construed as a potential conflict of interest.

## Publisher's note

All claims expressed in this article are solely those of the authors and do not necessarily represent those of their affiliated organizations, or those of the publisher, the editors and the reviewers. Any product that may be evaluated in this article, or claim that may be made by its manufacturer, is not guaranteed or endorsed by the publisher.
